# Reduced acute myocardial ischemia–reperfusion injury in IL-6-deficient mice employing a closed-chest model

**DOI:** 10.1007/s00011-016-0931-4

**Published:** 2016-03-02

**Authors:** Willeke M. C. Jong, Hugo ten Cate, André C. Linnenbank, Onno J. de Boer, Pieter H. Reitsma, Robbert J. de Winter, Coert J. Zuurbier

**Affiliations:** Laboratory of Experimental Intensive Care and Anesthesiology, Department of Anesthesiology, Academic Medical Center, University of Amsterdam, Meibergdreef 9, 1105 AZ Amsterdam, The Netherlands; Laboratory for Clinical Thrombosis and Hemostasis, Department of Internal Medicine, Maastricht University, Maastricht, The Netherlands; Faculty of Engineering Mathematics and Computer Science, Section Bioelectronics, Delft University of Technology, Mekelweg 4, 2628 CD Delft, The Netherlands; Department of Pathology, Academic Medical Center, University of Amsterdam, Meibergdreef 9, 1105 AZ Amsterdam, The Netherlands; Einthoven Laboratory for Experimental Vascular Medicine, Department of Thrombosis and Hemostasis, Leiden University Medical Center, Leiden, The Netherlands; Department of Cardiology, Academic Medical Center, University of Amsterdam, Meibergdreef 9, 1105 AZ Amsterdam, The Netherlands

**Keywords:** Inflammation, IL-6, Closed-chest model, Ischemia/reperfusion injury, Heart

## Abstract

**Objective and design:**

We examined the role of IL-6 in the temporal development of cardiac ischemia–reperfusion injury employing a closed-chest I/R model.

**Materials/methods:**

Infarction, local and systemic inflammation, neutrophil infiltration, coagulation and ST elevation/resolution were compared between wild-type (WT) and IL-6-deficient (IL-6^−/−^) mice after 1 h ischemia and 0, ½, 3, and 24 h reperfusion.

**Results:**

IL-6 deficiency reduced infarct size at 3 h reperfusion (28.8 ± 4.5 % WT vs 17.6 ± 2.5 % IL-6^−/−^), which reduction persisted and remained similar at 24 h reperfusion (25.1 ± 3.0 % WT vs 14.6 ± 4.4 % IL-6^−/−^). Serum Amyloid A was reduced at 24 h reperfusion only (57.5 ± 4.9 WT vs 24.8 ± 5.6 ug/ml IL-6^−/−^ mice). Cardiac cytokines (IL-6, IL-1β and TNFα) peaked at 3 h reperfusion, but IL-1β and TNFα levels were unaffected by IL-6 deficiency. Significant neutrophil influx was only detected at 24 h reperfusion and was similar for WT and IL-6^−/−^. Tissue factor peaked at 24 h reperfusion, whereas fibrin/fibrinogen peaked at 3 h reperfusion and was completely resolved at 24 h reperfusion; both coagulation factors were unaltered by IL-6 deficiency. Prolonged ST elevation was observed during ischemia that completely resolved for both genotypes at early reperfusion.

**Conclusions:**

The data suggest that, in the absence of major surgical intervention, IL-6 contributes to the development of infarct size in the early phase of reperfusion; this contribution did not depend on neutrophil influx, IL-1β and TNFα, tissue factor and fibrin.

## Introduction

Acute myocardial ischemia–reperfusion (I/R) is associated with an inflammatory response that may contribute to I/R-induced injury of the heart [1, 2]. One important pleiotropic inflammatory mediator that is released quickly upon cardiac I/R is interleukin (IL)-6 [3, 4]. Clinical studies have shown that chronically elevated IL-6 levels in blood are independently associated with acute coronary syndromes, suggesting an etiologic role for this cytokine in the process of myocardial injury [5, 6]. However, several pre-clinical animal studies have refuted IL-6 as major contributor to acute cardiac injury. Left ventricular remodeling and survival after permanent ischemia [7], left ventricular remodeling after 48 h I/R [8] or infarct size development after an acute episode of I/R were similar between wild-type and IL-6^−/−^ mice [9–11]. Recently, Samanta et al. have shown that IL-6 did contribute to adverse remodeling at 35 days following I/R [8]. However, these studies performed I/R immediately following the surgical preparative intervention of chest opening and puncturing of the heart to allow the ligation of the LAD. This surgical intervention significantly raises the baseline level of cytokines whereupon the effects of I/R-induced cytokine production are studied [12]. In a previous study we demonstrated that 75 % of the inflammatory cytokine response to acute I/R in such an open-chest model could be ascribed to the chest opening. In addition, the opening of the chest was associated with priming of the NLRP3 inflammasome, which priming was not observable in the closed-chest model [13]. Knowing that effects of inflammatory mediators are highly dependent on the actual concentration of the cytokine and the state of the innate immune system (e.g., [1]), it is, therefore, still unknown whether IL-6 affects acute cardiac injury in the absence of major surgery, i.e., in a closed-chest model of cardiac I/R. Such a closed-chest model better reflects the condition of acute cardiac syndromes as occurring in AMI patients and/or during PCI procedures, whereas the open-chest model better mirrors cardiac surgical procedures. Therefore, in the present study we examined to what extent IL-6 affects acute myocardial I/R injury in a closed-chest mouse model.

IL-6 belongs with tumor necrosis factor (TNF)α and IL-1β to the pro-inflammatory cytokines and may, therefore, contribute to increases in serum amyloid A (SAA) [14] in the systematic circulation and to neutrophil influx into the injured cardiac tissue [15]. In addition, it has been suggested that IL-6 contributes to activation of the coagulation system in experimental models of endotoxemia, due to the existence of cross-talk between inflammation and coagulation [16, 17]. Such an increased coagulation, when associated with microvascular thrombosis, is then a possible mechanism through which IL-6 can increase infarct size after myocardial ischemia–reperfusion [18]. Possible changes in coagulation may then impact the resolution of ischemia during reperfusion due to changes in no-flow regions within the heart, as reflected by a divergent ST-segment resolution [19, 20].

In this study we hypothesized that (1) IL-6 contributes to acute myocardial ischemia–reperfusion injury, and (2) it does so through alteration in inflammatory pathways and activation of the coagulation system. We expected to find smaller infarct sizes in IL-6 deficient mice due to a less pronounced pro-inflammatory and pro-coagulant effect. We, therefore, measured infarct sizes, tested pro-inflammatory cardiac cytokine levels and systemic SAA levels, used immunohistochemistry to evaluate neutrophil influx and activation of the coagulation system (tissue factor and fibrin/fibrinogen), and used ECG analysis as a surrogate marker of no-flow in a closed-chest mouse model [21] after 1 h ischemia and ½, 3 or 24 h reperfusion.

## Materials and methods

### Animals

**Fig. 1:**
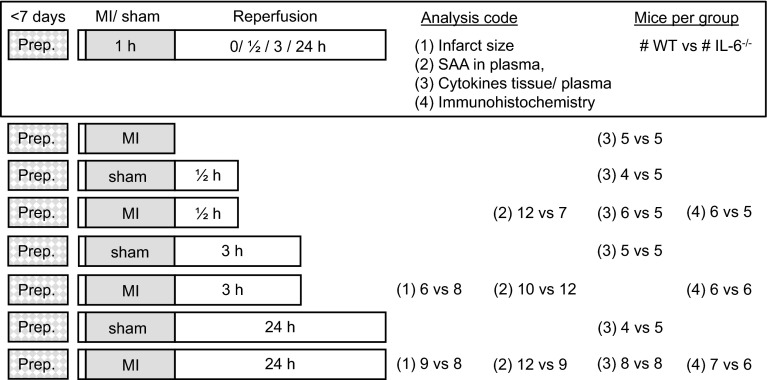
Flowchart with time frame, numbers of mice used and analyses performed

IL-6^−/−^ mice were originally generated by Kopf et al. [22]. Eight- to 10-week-old male IL-6^−/−^ mice (backcrossed onto a C57BL/6 background for at least 6 generations) and inbred C57BL/6 mice (for controls) (Jackson Laboratory; Bar Harbor, ME) were used in this study. They were housed in open topped cages at constant temperature (21–22 °C), humidity (50 ± 10 %) and light cycle (12 h dark–light). Rodent chow (AM-II 10 mm, Hope Farms, Woerden, The Netherlands) and drinking water were available ad libitum. Experiments were approved under number DIX 228 by the animal ethics committee of the Academic Medical Center, Amsterdam, The Netherlands, and comply with US National Institutes of Health or European Commission guidelines. The flowchart (Fig. 1) shows the number of mice in each analysis.

### Preparative surgery for I/R in closed chest

Stable anesthesia (with stable hemodynamic parameters) was induced by 10 ml FFM/kg BW i.p. (FFM: Fentanyl-Fluanisone-Midazolam) [23, 24]. Hypnorm^®^ (VetaPharma, Leeds, UK) contains 0.315 mg Fentanyl citrate/ml and 10 mg Fluanisone/ml. Midazolam^®^ (Actavis, Iceland) contains 5 mg Dormicum/ml. Both Hypnorm and Midazolam were 1:1 diluted in H_2_O prior to their 1:1 mixing. Anesthesia was maintained by bolus injections of ½ times the initial FFM volume. Paw withdrawal reflex and tail pinch were used to monitor the adequacy of anesthesia. After an induction time of 5 min, a preparative surgery was started as described previously [21]. Briefly, the skin of the throat was cut to visualize the trachea. The mice were intubated with an Intramedic PE-90 tube (Becton Dickinson, Sparks, Md), and volume controlled ventilated (CIV101 ventilator, Columbus Instruments, Ohio) with room air supplied with 40 % O_2_. Body temperature was maintained between 36.6 and 37.4 °C. Lateral thoracotomy was performed between the third and fourth rib, while the rectus abdominis and pectoralis were kept intact and put aside. The pericardium was locally opened, and a U-bended 8–0 prolene suture was put under the LAD approximately 2 mm lower than the tip of the left auricle, similarly as reported by Michael et al. [2] and Xu et al. [25]. The endings were tunneled in parallel through a 2 mm Intramedic PE-10 tube (Becton Dickinson, Sparks, Md) and separately brought outside between the adjacent ribs. The chest was closed under application of gentle pressure on both sides of the thorax to expel air, and the endings of the 8–0 prolene suture were hidden s.c. Post-operative analgesia was induced by 0.1 mg buprenorphine/kg s.c. (Temgesic^®^, Schering-Plough, Maarssen, The Netherlands). Sterile 0.5 ml 0.9 % saline i.p. was administered as fluid. Mice were allowed to recover in a temperature-controlled room of 30 °C for 1 day and at room temperature for >8 days.

### Ischemia–reperfusion

9–11 days after the preparative surgery, when the inflammatory reaction had subsided [12, 21] the mice were re-anesthetized with FFM, and allowed to freely breathe under 40 % oxygen-supplemented air. ECG (Einthoven lead I) was recorded by AgCL coated-silver needles. Output signals were amplified by a custom-made amplifier, sampled at 1 kHz, and band pass filtered at 100, 50 Hz and DC, and directly transferred to LabView5.0 (National Instruments, Austin, TX, USA). The skin was opened over the old scar, and the LAD (8–0 prolene) sutures were tightened for 1 h to achieve ischemia as verified by ECG recording. Release of the sutures, again confirmed by ECG, resulted in reperfusion. Mice were killed at different time points after 0, ½, 3, or 24 h reperfusion. Sham-treated mice had identical surgical procedures as the experimental group except that the sutures were not pulled. Mice in the 24 h reperfusion group were administered 0.1 mg buprenorphine/kg/s.c. (Temgesic^®^, Schering-Plough, Maarssen, The Netherlands) after 30 min reperfusion and housed in a temperature-controlled room of 30 °C. All mice were killed after reperfusion under FFM anesthesia.

### Blood sampling

After each experiment, the mice were heparinized via the tail vein (200 Units heparin (Leo Pharma, Breda, Netherlands) in 0.2 ml sterile saline) to prevent coagulation in the tissues [26]. Blood was collected from the vena cava into a plastic syringe containing 0.1 volume of 3.2 % sodium citrate. After centrifugation plasma samples were stored at −80 °C until further analysis.

### Infarct size

To identify the area at risk (AAR) and area of infarction (AOI) within the left ventricle of WT and IL-6^−/−^ mice after 3 and 24 h reperfusion, hearts were subjected to Evans blue and 2,3,5-triphenyl-tetrazolium chloride (TTC) staining [2]. Briefly, after exsanguination the chest was opened via medial section, on the right side of the septum. The sutures were tied to definitely occlude the LAD. A 23G1 Gauge blunted needle (Becton–Dickinson, Etten-Leur, The Netherlands) attached to a 2-ml syringe filled with 4 °C cardioplegic solution was cannulated through the aorta into the LV lumen and attached to the aorta. The heart was excised from the body and (retrogradely) infused with cardioplegia to rinse. Next, the needle was attached to a 1-ml syringe and the heart was infused with 50–100 μl 2 % Evans Blue (Sigma, St. Louis, MO, USA) to negatively mark the area at risk (AAR). The heart was cooled for several min at −20 °C after infusion of type VII low gelling temperature agarose (Sigma, St. Louis, MO, USA). The needle was removed and the heart was serially cut into transverse sections. The first section contained the suture around the LAD. To differentiate between the viable and necrotic areas of the myocardium within the AAR, the three 0.8 mm sections, the apex, and the base of the heart were soaked between Whatman filter paper and stained by 1.5 % TTC (Sigma-Aldrich, Steinheim, Germany) in phosphate buffer for 20 min at 37 °C. Each of the sections was weighed. Area of infarct (AOI), area at risk (AAR), and left ventricle (LV) (averaged from both sides of a slice) were assessed using computer-assisted planimetry (NIH imager, Scion Image, Meyer Instruments, Houston, TX, USA) to calculate volumes. Two observers blinded for genotypes and reperfusion times independently scored the images of the stained sections.

### Homogenate preparation

After exsanguinations, hearts were homogenized (1 vol) in Greenburger lysis buffer (300 mM NaCl, 15 mM Tris, 2 mM MgCl2, 2 mM Triton(X-100), Pepstatin A, Leupeptin, Aprotinin (20 ng/ml), pH 7.4) (9 vol). After centrifugation, the supernatant was frozen at −80 °C for cytokine measurements.

### Cytokines (in hearts and plasma), and SAA (in plasma)

After 24 h reperfusion, IL-6, IL-1β, and TNFα levels were in duplicate determined in plasma and in supernatants from control hearts, sham hearts, and from ischemic and non-ischemic parts (hearts were longitudinally split into two parts that contained the ischemic area and the area not at risk to distinguish the local inflammatory responses). Measurements of IL-6, IL-1β, and TNFα levels (detection level 0.50; 0.63; and 0.60 ng/mg, respectively) were performed using commercially available ELISA kits (R&D systems, Abingdon, UK).

SAA levels were singularly determined in remaining plasmas after 0, ½, 3, and 24 h reperfusion (detection level 55 ng/ml) (Biosource International, Inc. Camarillo, CA, USA).

### Tissue sampling for histology and immunohistochemistry

After ½, 3, and 24 h reperfusion, WT mice and IL-6^−/−^ were killed by exsanguinations under anesthesia (see paragraph "Blood sampling"). Hearts were taken from the chest, and transversally cut at the side where the suture was loosely saved after induction of reperfusion. The apex was removed and the mid-section was fixed in 4 % paraformaldehyde for 6 h at room temperature, and embedded in paraffin using automatic embedding equipment (Tissue Tek, Miles Scientific Inc., USA). 4 μm sections were obtained and every 30th slice was stained with hematoxylin and eosin, which resulted in 10–13 stained slices a heart.

For immunohistochemistry, the tissue sections were deparaffinized, rehydrated, and washed with water and PBS. Endogenous peroxidase activity was blocked by 1.5 % H_2_O_2_ in PBS for 30 min at room temperature, and washed in PBS. Non-specific binding sites were blocked with TENG-T [10 mM Tris, 5 mM EDTA, 0.15 M NaCl, 0.25 % gelatin, 0.05 % (vol/vol) Tween-20, pH 8.0] or 10 % normal goat serum (Dako, Glostrup, Denmark) for 30 min at RT, and then washed in PBS. Sections were incubated at 4 °C overnight with the different primary antibodies. The following antibodies were used: Ly6 for neutrophils (Pharmingen, San Diego, CA, USA), Tissue Factor (1:250 anti-tissue factor) and fibrin (Biotinylated goat anti-mouse fibrinogen (Accurate Chemical & Scientific Corporation, Westbury, NY) recognized fibrin/fibrinogen deposition). In each case, controls were treated with isotypic control IgG. Sections stained for Ly6G and fibrin were subsequently incubated with HRP conjugated swine anti rabbit immunoglobulins and enzyme activity was detected using 3,3′-diaminobenzidine-tetra-hydrochloride (DAB, Sigma-Aldrich, St. Louis, MO) or 3-amino-9-ethylcarbazole (AEC, Sigma-Aldrich) as a substrate. If the primary antibody was tissue factor, sections were sequentially incubated with biotinylated swine antirabbit immunoglobulins (Dako), strep-ABC^AP^ (Dako) and the Alkaline Phosphatase substrate kit I (Vector). The slides were mounted in Entallan or glycerin gelatin, respectively, after a slight hematoxylin (Ly6G), or no counterstaining (TF and fibrin/fibrinogen) and analyzed.

### Image analysis

The hematoxylin and eosin section with the largest visible I/R damage was the starting point to select 3 adjacent sections a heart for immunohistochemistry; The number of Ly-6G-positive cells, and the expression of tissue factor and fibrin/fibrinogen was investigated in the same areas and quantified by digital image analysis. In short, stained sections were scanned using the Philips IntelliSite Ultra Fast Scanner (Philips Digital Pathology Solutions, Best, The Netherlands). The mid-left ventricular free wall (0.33–1.2 mm [2]) was selected (endocardium and pericardium were always excluded) and a digital image was captured at 10× magnification. The resulting JPG files were further analyzed in ImageJ (US National Institutes of Health, Bethesda, MD). Ly6G-positive cells were manually counted using the ImageJ cell counter and expressed as number of neutrophils/mm [2]. Tissue factor and fibrin images were converted to 8-bit grayscale images and the immunopositivity was measured by setting an intensity threshold. The immunopositive area was expressed as a percentage of the total area.

### Analysis of the ST-segment

We limited our analysis of the ST-segment changes by studying randomly 20 WT and 20 IL-6^−/−^ mice. One WT mouse and two IL-6^−/−^ mice seemed to have disturbing electrical noise and were excluded from the data. The resulting ECG readings of 19 WT and 18 IL-6^−/−^ mice were averaged over 10 s recordings using software written in the Matlab (the Mathworks, USA) script language. Time alignment within a trace was performed using cross correlation with a template derived from a QRS complex. Because the morphology of the QRS complex changed as a result of the intervention it was not possible to align the differently shaped QRS complexes. Alignment of different traces was, therefore, done using the P-wave. Afterwards, the QRS complexes were aligned by hand. The amplitude of the ST-segment above the iso-electric line was measured 20 ms after the start of the QRS complex.

### Statistical analysis

All measurements are reported as mean ± SE. All data (except AOI/AAR, AAR/LV, and AOI/LV) were not normally distributed, and therefore we opted for non-parametric testing using the one-way analysis of Kruskal–Wallis on medians. A *P* value of less than 0.05 was considered statistically significant. Statistics were performed using the Prism software package (GraphPad, San Diego, CA).

## Results

**Fig. 2:**
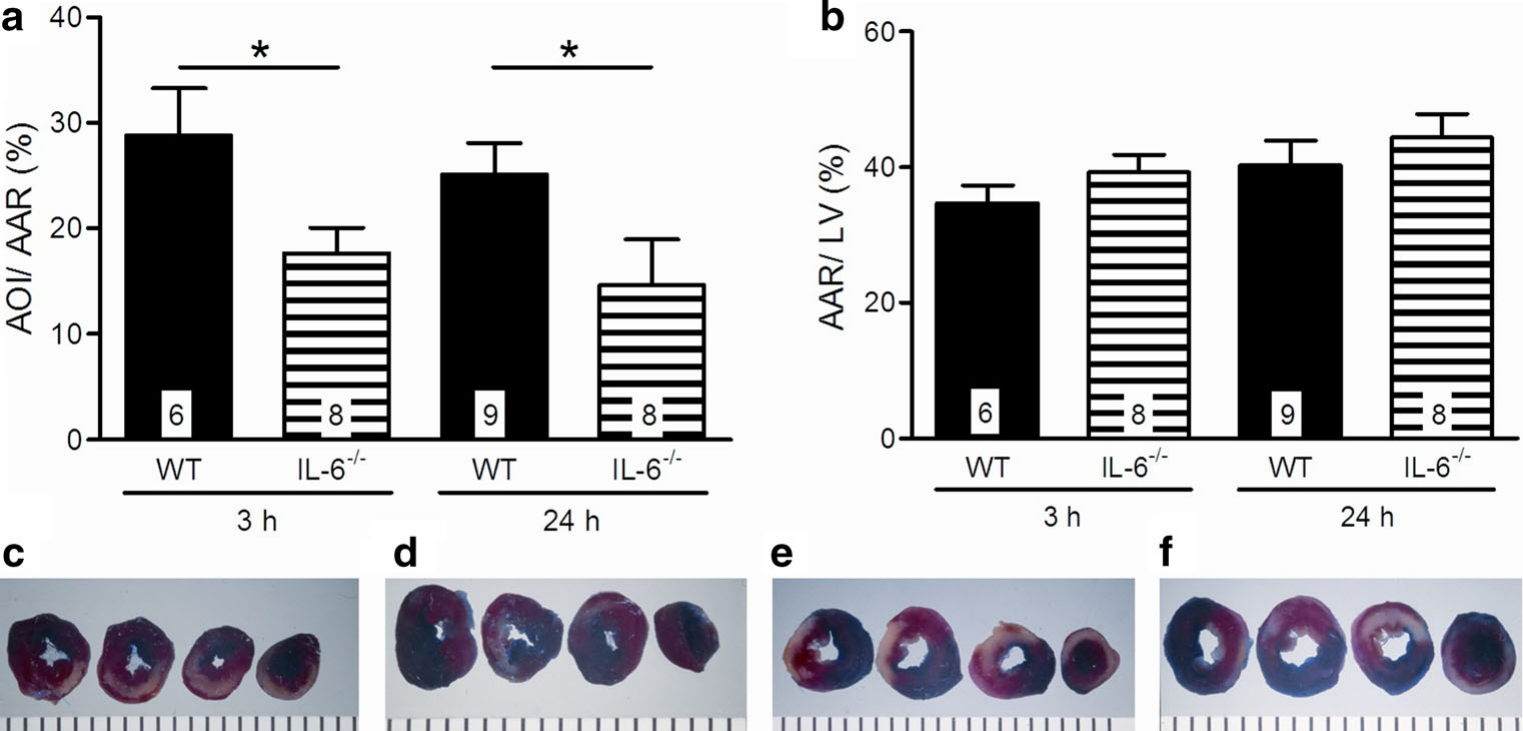
**a** Infarct sizes in WT mice and IL-6^−/−^ mice after 3 and 24 h reperfusion. **b** Area at risk sizes in WT mice and IL-6^−/−^ mice after 3 and 24 h reperfusion. Values are mean ± SEM; Black bars represent WT mice; Striped bars represent IL-6^−/−^ mice.* Numbers in the bars* indicate the number of analyzed samples. **c**–**f** Representative examples of Evans blue and TTC-stained hearts of, respectively, WT 3 h, IL-6^−/−^ 3 h, WT 24 h, and IL-6^−/−^ 24 h reperfusion

Infarct sizes as demarcated by TTC and Evans blue staining showed significantly smaller infarcts in IL-6^−/−^ mice when compared to WT mice after 3 h reperfusion: Fig. 2a shows infarct sizes of 28.8 ± 4.5 % in WT mice vs 17.6 ± 2.5 % in IL-6^−/−^ mice after 3 h reperfusion. No further change in infarct size with IL-6 ablation was observed at 24 h reperfusion, 25.1 ± 3.0 % in WT mice vs 14.6 ± 4.4 % in IL-6^−/−^ mice, indicating that IL-6-mediated effect on infarct development is effective during the first hours of reperfusion. The difference in infarct size between genotypes cannot be ascribed to differences in the area at risk (Fig. 2b). Figure 2c–f shows representative examples of TTC and Evans blue stainings.

**Fig. 3:**
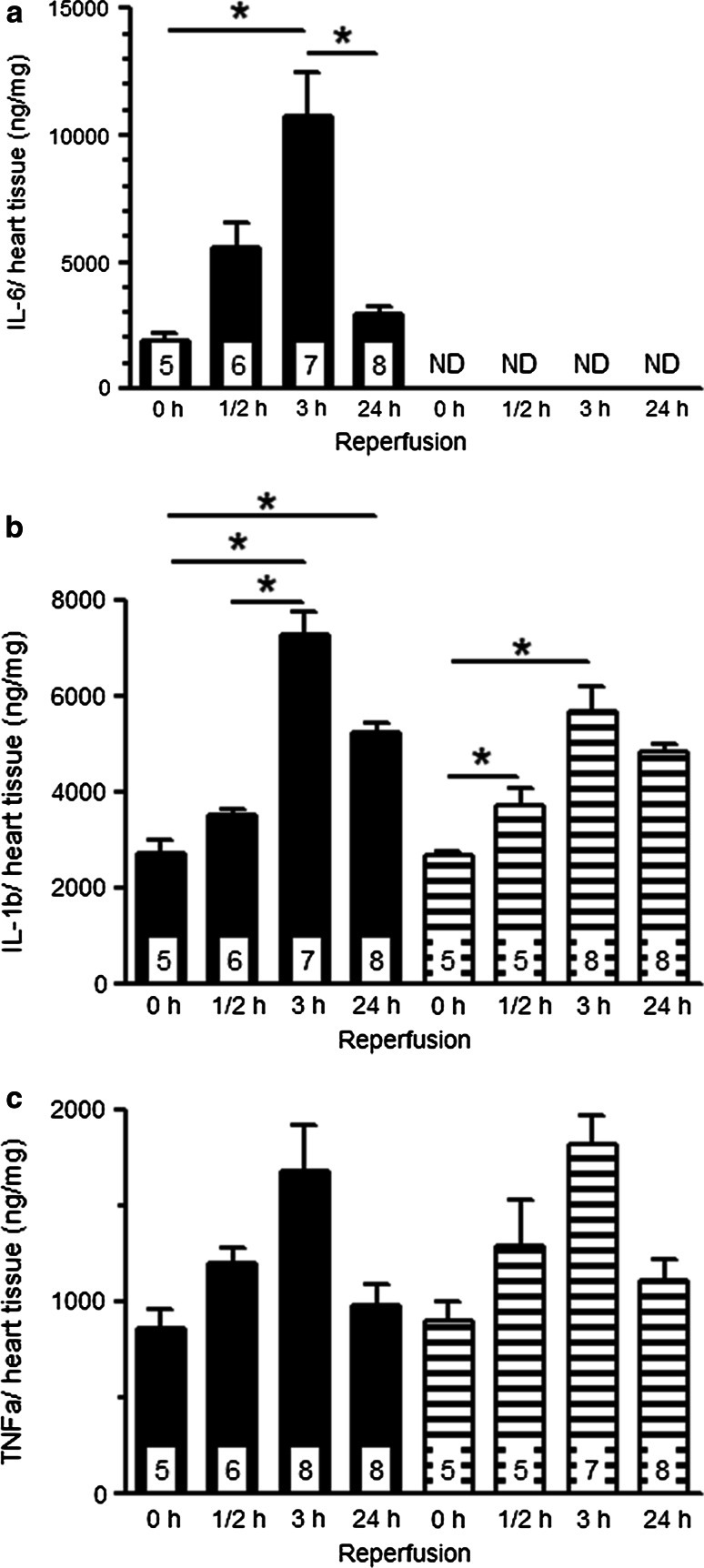
Cardiac cytokines, **a** IL-6, **b** IL-1β, and **c** TNFα in the ischemic part of the heart for WT and IL-6^−/−^ mice at 0, ½, 3, and 24 h reperfusion. Values are mean ± SEM; ND means undetectable; *p < 0.05; *black bars* represent WT mice; *striped bars* represent IL-6^−/−^ mice; *numbers* in the *bars* indicate the number of analyzed samples

To examine whether inflammation was constricted to the ischemic part of the heart only, we first compared the non-ischemic with the ischemic part of the heart for the ½ and 24 h reperfusion time points only. After ½ h reperfusion, cardiac IL-6 levels were already significantly elevated in the ischemic part of the heart as compared to the non-ischemic part in WT mice (5.6 ± 1.0 vs 1.7 ± 0.2 µg/mg; *n* = 6, respectively), indicating IL-6 to be a fast responder to cardiac ischemia (data not shown). After 24 h reperfusion cardiac IL-6 was only mildly elevated in the ischemic part, showing the transient behavior of IL-6 during I/R (ischemic 3.0 ± 0.2 vs non-ischemic 1.5 ± 0.1 µg/mg; *n* = 8) (data not shown). Thus, the lack of increase in IL-6 in the non-ischemic part of the heart throughout the experiment indicates that I/R-induced inflammation remains confined to the ischemic part of the heart. Figure 3 demonstrates levels of cardiac cytokines in the ischemic part of the heart for WT and IL-6^−/−^ mice at different times of reperfusion. IL-6 levels peaked at 3 h reperfusion, and were undetectable in IL-6^−/−^ hearts (Fig. 3a). A similar time pattern was observed for IL-1β with a peak at 3 h reperfusion (Fig. 3b). No differences in IL-1β were observed between WT and IL-6^−/−^ at any time point, suggesting that IL-6 is without effect on IL-1β levels. Cardiac TNFα also peaked at 3 h reperfusion, with no differences in TNFα levels between WT and IL-6^−/−^ mice at any time of reperfusion (Fig. 3c). Cardiac IL-6, IL-1β and TNFα levels in ischemic area and the non-ischemic area were not different in sham groups at 0, ½ and 24 h reperfusion (data not shown), indicating that the temporal changes in cytokines were due to the I/R intervention per se.

**Fig. 4:**
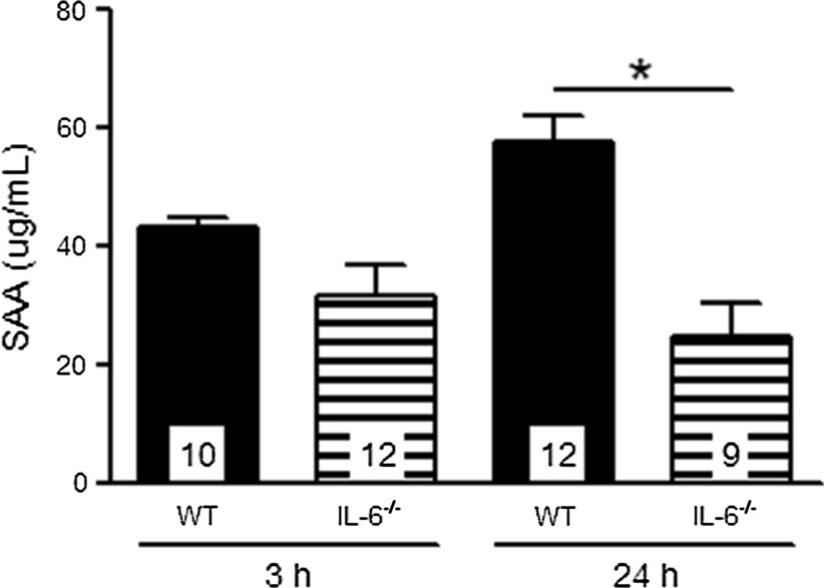
Plasma SAA levels in WT and IL-6^−/−^ mice after 3 and 24 h reperfusion. Values are mean ± SEM; *p < 0.05; *black bars* represent WT mice; *striped bars* represent IL-6^−/−^ mice; *numbers* in the *bars* indicate the number of analyzed samples

Next we examined whether the omission of IL-6 affected the acute phase protein response, by monitoring the production of SAA (produced by liver). Plasma SAA levels after 0 and ½ h reperfusion in WT (*n* = 4 and 12) and IL-6^−/−^ (*n* = 6 and 12) mice were undetectable. SAA levels increased significantly in WT mice after 3 h and 24 h reperfusion and were highest after 24 h reperfusion (Fig. 4). SAA was also increased at 3 h reperfusion in IL-6^−/−^ mice, however, in contrast to WT mice, no further increase in SAA was observed at 24 h reperfusion; resulting in significantly decreased SAA in IL-6^−/−^ mice at 24 h reperfusion. These data indicate that the production of the acute phase response protein SAA is partly, but not solely, dictated by the rise in IL-6.

**Fig. 5:**
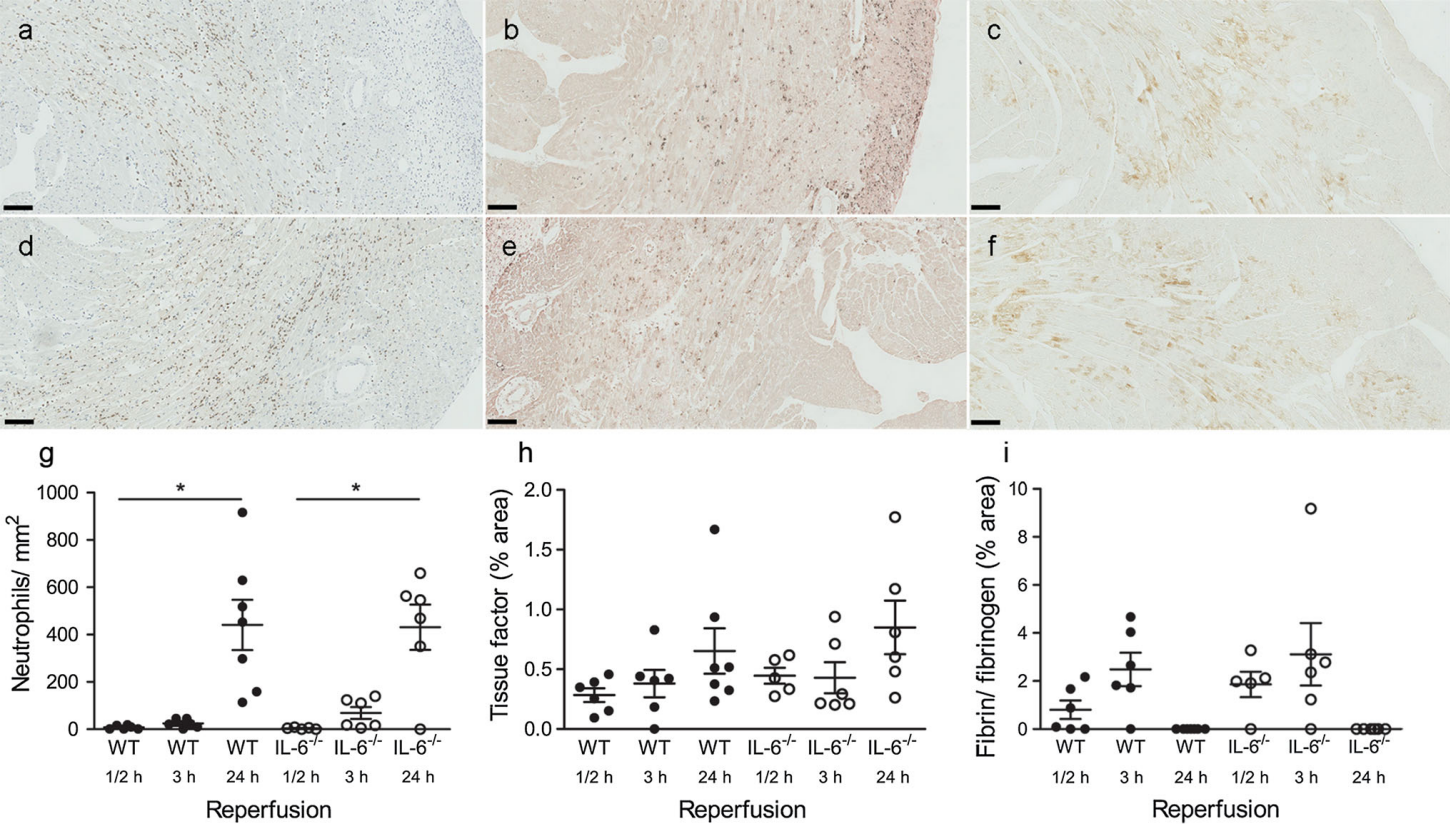
Immunohistochemical analysis of the left ventricular wall of WT (**a**–**c**) and IL6^−/−^ (**d**–**f**) mice after I/R injury. **a**, **d** Neutrophils (Ly6G) after 24 h reperfusion; **b**, **e** tissue factor after 24 h reperfusion; **c**, **f** fibrin/fibrinogen after 3 h reperfusion. *Bar* 100 µm. Quantitative analysis of neutrophils (**g**), tissue factor (**h**) and fibrin/fibrinogen (**i**) after ½, 3, and 24 h reperfusion. Values are mean ± SEM; *black dots* represent WT mice; *open dots* represent IL-6^−/−^ mice

Having established that IL-6 affected the acute development of cardiac infarction and the rise in the systemic inflammatory marker SAA, we now examined whether IL-6 plays a role in neutrophil influx and coagulation activation following cardiac I/R through immunohistochemical analysis (Fig. 5). Typical examples of staining for neutrophil, tissue factor and fibrin/fibrinogen for WT and IL-6^−/−^ are demonstrated in Fig. 5a–f. The influx of neutrophils in our model of closed-chest cardiac I/R shows a later response as compared to the cytokine response. Influx of neutrophils at ½ h was non-detectable, and only very minimal present at 3 h reperfusion. At 24 h reperfusion there was a significantly increased influx of neutrophils, however, this influx was similar between WT and the IL-6^−/−^ mice (Fig. 5g). Tissue factor was already detectable at ½ h of reperfusion and demonstrated a non-significant increase with prolongation of reperfusion time, without any significant effect of IL-6 on this development of tissue factor during reperfusion (Fig. 5h). Finally, immunostaining for fibrin/fibrinogen also revealed the early presence of this coagulation factor that peaked at 3 h reperfusion, but completely disappeared at 24 h reperfusion, with no differences between WT and IL-6^−/−^ (Fig. 5i). Data suggest that the cytokine IL-6 is not involved in the early recruitment of neutrophils or the activation of the coagulation system in the early reperfusion phase when employing a closed-chest model of cardiac I/R.

**Fig. 6:**
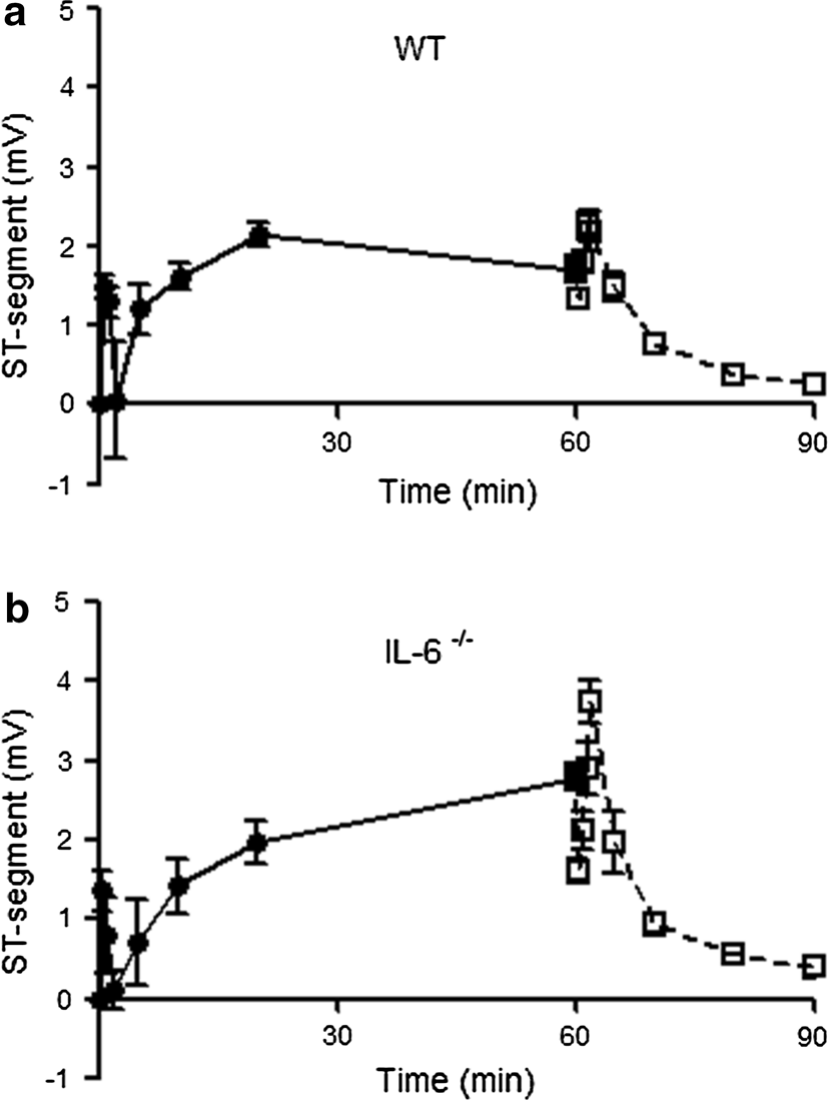
Averaged ST-segment (mV) during 60 min ischemia (*uninterrupted line*) and during the early reperfusion phase (*interrupted line*) for **a** WT mice (*n* = 19) and **b** IL-6^−/−^ mice (*n* = 18)

Finally, we applied ECG recording during cardiac I/R to monitor ST elevation as surrogate marker of degree of ischemia and successful and complete reperfusion. Figure 6 shows a summary of the averaged ST-segment elevation during ischemia, and its resolution during the early reperfusion phase for WT (Fig. 6a) and IL-6^−/−^ (Fig. 6b) mice. Pulling of the sutures induced an immediate ST-segment elevation that peaked after 30 s. The ST-segment elevation showed a short lasting normalization at 2 min ischemia and increased for a second time to reach its maximum after 20 min ischemia. The elevation persisted during the remaining ischemic period. Releasing of the sutures induced a short episode of partial ST-segment resolution that lasted approximately 30 s. Next the ST-segments were again elevated at 1½ min at a level that was higher than during ischemia. This temporal increase disappeared within 5 min and the ST-segment resolution was complete after 30 min for both WT and IL-6^−/−^ mice. In none of the mice a secondary rise or re-elevation in ST-segments was observed during reperfusion.

## Discussion

In this study we have demonstrated that IL-6 is one of the mediating factors that contributes to the development of cardiac infarct size during the early period (<1 day) of reperfusion, following an injurious period of cardiac ischemia in the absence of surgical stress. In addition, our data suggest that the observed IL-6 effects on acute cardiac infarction are not mediated through changes in IL-1β, TNFα, neutrophil influx or an altered activation of the tissue factor-thrombin pathway.

### IL-6 deficiency and cardiac infarct: closed-chest versus open-chest cardiac I/R models

In our closed-chest model, the absence of IL-6 resulted in smaller infarcts. These results are in contrast to previous studies employing the open-chest model of cardiac I/R, reporting no effect of IL-6 deficiency on infarct size [9, 10]. Previous studies have demonstrated [12, 13] that the major difference between these models is the enhanced activation of inflammatory pathways; i.e., the surgical stress of chest opening resulted in inflammatory priming. We demonstrated that >75 % of inflammation in the open-chest model is due to surgery, and not to the actual cardiac I/R [13]. As such, the open-chest model mimics the clinical condition of cardiac I/R during CABG procedures, whereas the closed-chest model mimics the clinical condition of cardiac I/R during PCI or thrombolytic procedures. Thus, these data indicate that under conditions of an already activated inflammatory background (due to e.g., surgery in the open-chest model, sepsis, etc.), the role of a single acute inflammatory responder such as IL-6 on acute reperfusion injury is not detectable. Although open-chest studies suggest that inflammatory pathways/mediators other than IL-6 become more critical for modulating cardiac I/R injury in these conditions (e.g., the Nlrp3 inflammasome [13], TLR4 [27, 28]), further studies are necessary to decipher the precise molecular mechanisms explaining the different outcome between the open-chest and closed-chest models. Infarct sizes in closed-chest models are also usually much smaller (5–30 % of risk area; present study and Durgan et al. [29]) than infarct sizes in open-chest studies (35–60 % of risk area), despite the fact that in these closed-chest models a longer duration of ischemia (45–60 min) is used as compared to the open-chest models (30 min ischemia). The decreased inflammation in the closed-chest models is thus associated with a smaller infarct size as compared to open-chest models, supporting the concept that inflammation contributes to the acute infarct development upon reperfusion.

Theoretically, the inflammatory response caused by the preparative surgery could have a preconditioning effect. However, since such preconditioning has an early window (2–3 h) and a delayed window (which begins 12–24 h later and lasts 3–4 days) of protection [30] that is much shorter than the 9–11 days pause we had chosen, it is unlikely that preconditioning could have affected our I/R results in the closed-chest model.

### Inflammatory mediators and I/R-induced acute cardiac infarction

Inflammation plays a modulating role in cardiac ischemia–reperfusion, both in the early reperfusion phase during infarct development and in the post-infarct phase during cardiac remodeling [31, 32]. Early responder cytokines such as TNFα and IL-6, localized in resident mast cells and endothelial cells, are quickly released and increased in expression upon the early moments of cardiac I/R. However, their role on modulating infarct size is likely critically dependent on the precise pathophysiological conditions present. TNFα knockout mice are reported to have reduced or unaltered infarct size in the isolated heart or open-chest model [32]. IL-6^−/−^ mice are now also reported to have reduced (present study) or unaltered infarct size [9, 10]. Deletion of IL-6 was also reported to protect against acute kidney I/R [33]. It is becoming increasingly clear that these cytokines are all part of a larger network of interacting cytokines that depending on context (e.g., background inflammation, timing in I/R processes) can activate or inhibit cell death/survival signaling cascades. One, often ignored, important context characteristic, concerns the duration of the ischemic episode; we previously demonstrated this for the cardioprotective effects of folic acid: folic acid protected against 25 min ischemia, but not against 35 min ischemia [34]. Similarly, it was shown that deletion of the macrophage migration inhibitory factor protected hearts from 60 min ischemia, but trended to increase I/R injury with much shorter ischemia [35]. It is, therefore, also possible that the protection we now describe with IL-6 deletion following 60 min ischemia is still commensurate with findings that report no effect of IL-6 deletion following only 30 min ischemia [9, 10]. Literature indicates that mechanisms contributing to I/R injury differ depending on the ischemic duration, such that the efficacy of factors affecting specific mechanisms also depends on ischemia duration.

For TNFα it has been reported that this cytokine can indeed directly contribute to cell death [36], however, no such data can be found for IL-6. It has been reported that IL-6 induces mitochondrial dysfunction (decreased ATP production, increased ROS production) in adipocytes [37] but, although mitochondria are crucial mediators of cardiac I/R injury, it is unknown whether similar IL-6 effects are obtained in myocardial tissue. We did find decreased amounts of the acute phase response protein SAA in IL-6^−/−^ mice. It has been shown that SAA can function as an agonist for the innate immune receptors TLR2/4 [38, 39], which receptors are part of the signaling cascades that contribute to acute cardiac infarction [27, 28, 40]. Thus, it is possible that IL-6 contributes to infarct development indirectly through activation of TLR cellular pathways.

### IL-6 effects on infarct cannot be explained by neutrophils and coagulation

In an attempt to further examine factors that contribute to myocardial injury in WT and IL-6^−/−^ mice, we examined neutrophil influx by histology. It is known from literature that the induction and release of IL-6 induces the expression of intercellular adhesion molecule-1 (ICAM-1) on the myocyte surface in the viable border zone of the infarct [41]. The etiologic role of released IL-6 in stimulation of myocyte ICAM-1 was previously demonstrated by others [42], and binding of neutrophils to ICAM-1 is considered to be a causal factor in myocyte injury after ischemia [15, 43, 44]. Although we did not quantify ICAM-1 expression ourselves, neutrophils were uniformly distributed throughout the previous ischemic area both in WT and IL-6^−/−^ mice; the influx in time and the distribution were equal in WT and IL-6^−/−^ mice suggesting that IL-6 is not important for the early recruitment of neutrophils to reperfused ischemic tissue. This can be taken as evidence that the causal pathway IL-6-ICAM-1-neutrophil is not operational in the present model; alternatively, if operational in WT mice, it apparently can be bypassed easily by other cytokines in IL-6^−/−^ mice. In fact, TNFα was shown previously to induce ICAM-1 and subsequent neutrophil influx instead of IL-6 or IL-1β [45]. Since TNFα levels were similar between WT and IL-6^−/−^ mice within the first 24 h reperfusion in our study, this could well explain the fact that neutrophil influxes appeared to be independent of IL-6. Most importantly, the similar influx of neutrophils between WT and IL-6^−/−^ mice negates that neutrophils can explain the reduced infarction observed in the IL-6^−/−^ mice. Moreover, the difference in the time course between infarction (complete at 3 h reperfusion, no further increase with 24 h reperfusion) and influx of neutrophils (hardly detectable at 3 h reperfusion, only at 24 h reperfusion) also makes it rather unlikely that neutrophils contributed to cardiac infarction in our model. This is commensurate with current thinking that I/R-induced infarction mostly takes place during the first 30 min reperfusion, and is dictated by mechanisms such as opening of the mitochondrial transition pore, activation of calpains and hypercontractility [46, 47].

Another element in myocardial injury that may be affected by different IL-6 levels relates to coagulation activation. IL-6 is known to be an important agonist of monocytic tissue factor induction in humans and primates, either directly, or after endotoxin stimulation [16, 17], thus we expected a role of IL-6 in inducing myocardial tissue factor and subsequent fibrin formation. In addition, previous studies using an antibody against tissue factor, active protein C or active site inhibited factor VIIa demonstrated reduction in infarcted area, thus demonstrating a possible causal relationship between coagulation and ischemic myocardial injury [18, 48, 49]. However, it has recently been suggested that these anticoagulants may also directly activate cytoprotective signaling independent of coagulation inhibition [50, 51].

We were unable to detect any differences in the presence of tissue factor or fibrin at any time of reperfusion between WT and IL-6^−/−^ mice. A possible reason as to why IL-6 does not seem to affect coagulation parameters may be related to the rather low and short-term activation of coagulation in our closed-chest cardiac I/R model. Although there is a trend in increased tissue factor at 24 h of reperfusion, the fibrin data indicate complete disappearance of fibrin at 24 h reperfusion. The fast disappearance of fibrin suggests resolution of coagulation at 24 h of reperfusion. This is also indirectly supported by our ECG monitoring. The ischemia-induced ST elevation was quickly and equally resolved during reperfusion in both WT and IL-6^−/−^ mice. It is, therefore, likely that IL-6 exerts its detrimental effects on acute infarct development through mechanisms other than coagulation activation or neutrophil action.

In conclusion, our data indicate that IL-6 contributes to the development of infarction, in a closed-chest model of cardiac I/R that is associated with attenuated inflammation as compared to open-chest models. The beneficial effects of IL-6 deletion on infarction cannot be explained by modification of other inflammatory mediators, neutrophil influx or coagulation activation. The effects of IL-6 are apparent within the first 3 h of reperfusion, suggesting a more direct, cellular signaling effect of IL-6 on the cellular mechanism that causes I/R-induced cell death. The present findings merit a further investigation into these direct molecular mechanisms of IL-6 related to cardiac I/R injury.

## References

[1] Frangogiannis NG (2014). The inflammatory response in myocardial injury, repair, and remodelling. Nat Rev Cardiol.

[2] Michael LH, Entman ML, Hartley CJ, Youker KA, Zhu J, Hall SR, Hawkins HK, Berens K, Ballantyne CM (1995). Myocardial ischemia and reperfusion: a murine model. Am J Physiol.

[3] Gwechenberger M, Mendoza LH, Youker KA, Frangogiannis NG, Smith CW, Michael LH, Entman ML (1999). Cardiac myocytes produce interleukin-6 in culture and in viable border zone of reperfused infarctions. Circulation.

[4] Kukielka GL, Smith CW, Manning AM, Youker KA, Michael LH, Entman ML (1995). Induction of interleukin-6 synthesis in the myocardium. Potential role in postreperfusion inflammatory injury. Circulation.

[5] Empana JP, Jouven X, Canoui-Poitrine F, Luc G, Tafflet M, Haas B, Arveiler D, Ferrieres J, Ruidavets JB, Montaye M, Yarnell J, Morange P, Kee F, Evans A, Amouyel P, Ducimetiere P (2010). C-reactive protein, interleukin 6, fibrinogen and risk of sudden death in European middle-aged men: the PRIME study. Arterioscler Thromb Vasc Biol.

[6] Manten A, de Winter RJ, Minnema MC, ten Cate H, Lijmer JG, Adams R, Peters RJ, van Deventer SJ (1998). Procoagulant and proinflammatory activity in acute coronary syndromes. Cardiovasc Res.

[7] Fuchs M, Hilfiker A, Kaminski K, Hilfiker-Kleiner D, Guener Z, Klein G, Podewski E, Schieffer B, Rose-John S, Drexler H (2003). Role of interleukin-6 for left ventricular remodeling and survival after experimental myocardial infarction. Faseb J.

[8] Samanta A, Cheng G, Davani A, Girgis M, Chen L, Choksi K, Zhao L, Vincent RJ, Hauptman J, Dawn B. Abstract 15693: Genetic Deletion of Interleukin-6 Attenuates Left Ventricular Dysfunction and Remodeling After a Reperfused Myocardial Infarction. Circulation. 2014;130(Suppl 2):A15693.

[9] Kaminski KA, Kozuch M, Bonda TA, Stepaniuk MM, Waszkiewicz E, Chyczewski L, Musial WJ, Winnicka MM (2009). Effect of interleukin 6 deficiency on the expression of Bcl-2 and Bax in the murine heart. Pharmacol Rep.

[10] Dawn B, Xuan YT, Guo Y, Rezazadeh A, Stein AB, Hunt G, Wu WJ, Tan W, Bolli R (2004). IL-6 plays an obligatory role in late preconditioning via JAK-STAT signaling and upregulation of iNOS and COX-2. Cardiovasc Res.

[11] McGinnis GR, Ballmann C, Peters B, Nanayakkara G, Roberts M, Amin R, Quindry JC (2015). Interleukin-6 mediates exercise preconditioning against myocardial ischemia reperfusion injury. Am J Physiol Heart Circ Physiol.

[12] Nossuli TO, Lakshminarayanan V, Baumgarten G, Taffet GE, Ballantyne CM, Michael LH, Entman ML (2000). A chronic mouse model of myocardial ischemia–reperfusion: essential in cytokine studies. Am J Physiol Heart Circ Physiol.

[13] Jong WMC, Leemans JC, Weber NC, Juffermans NP, Schultz MJ, Hollmann MW, Zuurbier CJ (2014). Nlrp3 plays no role in acute cardiac infarction due to low cardiac expression. Int J Cardiol.

[14] Siewert E, Bort R, Kluge R, Heinrich PC, Castell J, Jover R (2000). Hepatic cytochrome P450 down-regulation during aseptic inflammation in the mouse is interleukin 6 dependent. Hepatology.

[15] Hawkins HK, Entman ML, Zhu JY, Youker KA, Berens K, Dore M, Smith CW (1996). Acute inflammatory reaction after myocardial ischemic injury and reperfusion. Development and use of a neutrophil-specific antibody. Am J Pathol.

[16] Stouthard JM, Levi M, Hack CE, Veenhof CH, Romijn HA, Sauerwein HP, van der Poll T (1996). Interleukin-6 stimulates coagulation, not fibrinolysis, in humans. Thromb Haemost.

[17] van der Poll T, Levi M, Hack CE, ten Cate H, van Deventer SJ, Eerenberg AJ, de Groot ER, Jansen J, Gallati H, Buller HR (1994). Elimination of interleukin 6 attenuates coagulation activation in experimental endotoxemia in chimpanzees. J Exp Med.

[18] Erlich JH, Boyle EM, Labriola J, Kovacich JC, Santucci RA, Fearns C, Morgan EN, Yun W, Luther T, Kojikawa O, Martin TR, Pohlman TH, Verrier ED, Mackman N (2000). Inhibition of the tissue factor-thrombin pathway limits infarct size after myocardial ischemia–reperfusion injury by reducing inflammation. Am J Pathol.

[19] Gehrmann J, Frantz S, Maguire CT, Vargas M, Ducharme A, Wakimoto H, Lee RT, Berul CI (2001). Electrophysiological characterization of murine myocardial ischemia and infarction. Basic Res Cardiol.

[20] Li RA, Leppo M, Miki T, Seino S, Marban E (2000). Molecular basis of electrocardiographic ST-segment elevation. Circ Res.

[21] Jong WMC, Reitsma PH, ten Cate H, de Winter RJ (2003). Modified two-step model for studying the inflammatory response during myocardial ischemia and reperfusion in mice. Comp Med.

[22] Kopf M, Baumann H, Freer G, Freudenberg M, Lamers M, Kishimoto T, Zinkernagel R, Bluethmann H, Kohler G (1994). Impaired immune and acute-phase responses in interleukin-6-deficient mice. Nature.

[23] Jong WMC, Zuurbier CJ, De Winter RJ, Van Den Heuvel DAF, Reitsma PH, Ten Cate H, Ince C (2002). Fentanyl fluanisone midazolam Combination Results in More Stable Hemodynamics than Does Urethane a-chloralose and 2,2,2-tribromoethanol in Mice. Contemp Top Lab Anim Sci.

[24] Zuurbier CJ, Koeman A, Houten SM, Hollmann MW, Florijn WJ (2014). Optimizing anesthetic regimen for surgery in mice through minimization of hemodynamic, metabolic, and inflammatory perturbations. Exp Biol Med (Maywood).

[25] Xu Z, Alloush J, Beck E, Weisleder N. A murine model of myocardial ischemia–reperfusion injury through ligation of the left anterior descending artery. Journal of visualized experiments JoVE. 2014; (86). 10.3791/51329 PMC4080806 24747599

[26] Weiler-Guettler H, Christie PD, Beeler DL, Healy AM, Hancock WW, Rayburn H, Edelberg JM, Rosenberg RD (1998). A targeted point mutation in thrombomodulin generates viable mice with a prethrombotic state. J Clin Investig.

[27] Chong AJ, Shimamoto A, Hampton CR, Takayama H, Spring DJ, Rothnie CL, Yada M, Pohlman TH, Verrier ED (2004). Toll-like receptor 4 mediates ischemia/reperfusion injury of the heart. J Thorac Cardiovasc Surg.

[28] Oyama J, Blais C, Liu X, Pu M, Kobzik L, Kelly RA, Bourcier T (2004). Reduced myocardial ischemia–reperfusion injury in toll-like receptor 4-deficient mice. Circulation.

[29] Durgan DJ, Pulinilkunnil T, Villegas-Montoya C, Garvey ME, Frangogiannis NG, Michael LH, Chow CW, Dyck JR, Young ME (2010). Short communication: ischemia/reperfusion tolerance is time-of-day-dependent: mediation by the cardiomyocyte circadian clock. Circ Res.

[30] Fisher SG, Marber MS (2002). An in vivo model of ischaemia-reperfusion injury and ischaemic preconditioning in the mouse heart. J Pharmacol Toxicol Methods.

[31] Christia P, Frangogiannis NG (2013). Targeting inflammatory pathways in myocardial infarction. Eur J Clin Invest.

[32] Schulz R (2008). TNFalpha in myocardial ischemia/reperfusion: damage vs. protection. J Mol Cell Cardiol.

[33] Patel NSA, Chatterjee PK, Di Paola R, Mazzon E, Britti D, De Sarro A, Cuzzocrea S, Thiemermann C (2005). Endogenous interleukin-6 enhances the renal injury, dysfunction, and inflammation caused by ischemia/reperfusion. J Pharmacol Exp Ther.

[34] Zuurbier CJ, Heinen A, Koeman A, Stuifbergen R, Hakvoort TB, Weber NC, Hollmann MW (2014). Cardioprotective efficacy depends critically on pharmacological dose, duration of ischaemia, health status of animals and choice of anaesthetic regimen: a case study with folic acid. J Transl Med.

[35] Gao XM, Liu Y, White D, Su Y, Drew BG, Bruce CR, Kiriazis H, Xu Q, Jennings N, Bobik A, Febbraio MA, Kingwell BA, Bucala R, Fingerle-Rowson G, Dart AM, Morand EF, Du XJ (2011). Deletion of macrophage migration inhibitory factor protects the heart from severe ischemia–reperfusion injury: a predominant role of anti-inflammation. J Mol Cell Cardiol.

[36] Marshall KD, Baines CP (2014). Necroptosis: is there a role for mitochondria?. Front Physiol.

[37] Ji C, Chen X, Gao C, Jiao L, Wang J, Xu G, Fu H, Guo X, Zhao Y (2011). IL-6 induces lipolysis and mitochondrial dysfunction, but does not affect insulin-mediated glucose transport in 3T3-L1 adipocytes. J Bioenerg Biomembr.

[38] Cheng N, He R, Tian J, Ye PP, Ye RD (2008). Cutting edge: TLR2 is a functional receptor for acute-phase serum amyloid A. J Immunol.

[39] Sandri S, Rodriguez D, Gomes E, Monteiro HP, Russo M, Campa A (2008). Is serum amyloid A an endogenous TLR4 agonist?. J Leukoc Biol.

[40] Kim SC, Ghanem A, Stapel H, Tiemann K, Knuefermann P, Hoeft A, Meyer R, Grohe C, Knowlton AA, Baumgarten G (2007). Toll-like receptor 4 deficiency: smaller infarcts, but no gain in function. BMC Physiol.

[41] Frangogiannis NG, Lindsey ML, Michael LH, Youker KA, Bressler RB, Mendoza LH, Spengler RN, Smith CW, Entman ML (1998). Resident cardiac mast cells degranulate and release preformed TNF-alpha, initiating the cytokine cascade in experimental canine myocardial ischemia/reperfusion. Circulation.

[42] Entman ML, Youker K, Shoji T, Kukielka G, Shappell SB, Taylor AA, Smith CW (1992). Neutrophil induced oxidative injury of cardiac myocytes. A compartmented system requiring CD11b/CD18-ICAM-1 adherence. J Clin Invest.

[43] Youker K, Smith CW, Anderson DC, Miller D, Michael LH, Rossen RD, Entman ML (1992). Neutrophil adherence to isolated adult cardiac myocytes. Induction by cardiac lymph collected during ischemia and reperfusion. J Clin Invest.

[44] Metzler B, Mair J, Lercher A, Schaber C, Hintringer F, Pachinger O, Xu Q (2001). Mouse model of myocardial remodelling after ischemia: role of intercellular adhesion molecule-1. Cardiovasc Res.

[45] Ikeda U, Ikeda M, Kano S, Shimada K (1994). Neutrophil adherence to rat cardiac myocyte by proinflammatory cytokines. J Cardiovasc Pharmacol.

[46] Garcia-Dorado D, Ruiz-Meana M, Piper HM (2009). Lethal reperfusion injury in acute myocardial infarction: facts and unresolved issues. Cardiovasc Res.

[47] Halestrap AP, Richardson AP (2015). The mitochondrial permeability transition: a current perspective on its identity and role in ischaemia/reperfusion injury. J Mol Cell Cardiol.

[48] Loubele ST, Spek CA, Leenders P, van Oerle R, Aberson HL, Hamulyak K, Ferrell G, Esmon CT, Spronk HM, ten Cate H (2009). Activated protein C protects against myocardial ischemia/reperfusion injury via inhibition of apoptosis and inflammation. Arterioscler Thromb Vasc Biol.

[49] Loubele ST, Spek CA, Leenders P, van Oerle R, Aberson HL, van der Voort D, Hamulyak K, Petersen LC, Spronk HM, ten Cate H (2009). Active site inhibited factor VIIa attenuates myocardial ischemia/reperfusion injury in mice. J Thromb Haemost.

[50] Griffin JH, Zlokovic BV, Mosnier LO (2015). Activated protein C: biased for translation. Blood.

[51] Cohen MV, Downey JM (2015). Signalling pathways and mechanisms of protection in pre- and postconditioning: historical perspective and lessons for the future. Br J Pharmacol.

